# Prostate Cancer Skeletal Metastasis: A Spontaneous Evolution from Osteolytic to Osteoblastic Morphology without Treatment

**DOI:** 10.1055/s-0043-1777697

**Published:** 2023-12-26

**Authors:** Ismaheel O. Lawal, Mehmet A. Bilen, Raghuveer K. Halkar, Ashesh B. Jani, David M. Schuster

**Affiliations:** 1 Department of Radiology and Imaging Sciences, Emory University, Atlanta, Georgia, United States; 2 Department of Nuclear Medicine, University of Pretoria, Pretoria, South Africa; 3 Department of Hematology and Medical Oncology, Winship Cancer Institute of Emory University, Atlanta, Georgia, United States; 4 Department of Radiation Oncology, Winship Cancer Institute of Emory University, Atlanta, Georgia, United States

**Keywords:** ^18^ F-fluciclovine PET/CT, ^18^ F-rhPSMA PET/CT, prostate cancer, biochemical recurrence, skeletal metastasis progression

## Abstract

Skeletal metastases due to prostate cancer (PCa) are more commonly osteoblastic than osteolytic. In the rarer cases of osteolytic skeletal metastasis of PCa, transition to osteoblastic phenotype occurs following treatment, which indicates successful healing. In this report, we present a case of spontaneous osteolytic to osteoblastic evolution of PCa skeletal metastasis without treatment in a patient with recurrence of PCa. Our patient is a 59-year-old male who had a robotic radical prostatectomy in July 2014 for a T2c adenocarcinoma of the prostate gland (Gleason score = 4 + 3). He had adjuvant pelvic radiotherapy in January 2015 due to prostate-specific antigen (PSA) persistence. PSA began to rise in October 2015. An ^18^ F-fluciclovine positron emission tomography/computed tomography (PET/CT) scan obtained in June 2017 at a PSA of 0.5 ng/mL was negative. Repeat ^18^ F-fluciclovine PET/CT of February 2020 at PSA of 3.72 ng/mL showed prostate bed recurrence and a nonavid osteolytic left inferior pubic ramus lesion. 18F radiohybrid prostate-specific membrane antigen ( ^18^ F-rhPSMA) PET/CT scan of August 2020 performed as part of an ongoing clinical trial confirmed local prostate bed recurrence with a low-grade radiotracer uptake in the osteolytic left inferior pubic ramus bone lesion. Without salvage therapy, ^18^ F-fluciclovine PET/CT of October 2020 and March 2022 shows progressive sclerosis in the left pubic ramus lesion. An osteolytic to osteoblastic transition of a bone lesion as shown in this patient calls for a rethink in our understanding of untreated PCa skeletal metastasis progression. This case provides novel insight into the understanding of the temporal evolution of skeletal metastasis and calls for further research.

## Introduction

Radical prostatectomy is a recommended treatment option for patients with localized prostate cancer (PCa). 1 Radical prostatectomy is very effective for tumor eradication. However, disease recurrence heralded by a rise in serum prostate-specific antigen (PSA) occurs on long-term follow-up. 2 Imaging is critical for guiding salvage therapy of recurrent PCa. Traditionally, imaging of the recurrence of PCa has been done with conventional imaging applying combined radionuclide bone scan and computed tomography (CT) of the abdomen and pelvis. These conventional imaging modalities have limited diagnostic sensitivity at low PSA levels characteristic of early PCa recurrence. Early detection of the site of PCa recurrence and prompt institution of salvage therapy improves patients' survival. 3 Novel radionuclide imaging probes that target PCa-expressed targets have been developed for the improved detection of early recurrence of PCa. The most successful radiopharmaceuticals for the improved detection of PCa recurrence target amino acid trapping and the over-expression of prostate-specific membrane antigen (PSMA) by the PCa cells. 4 5 These radiopharmaceuticals have superior lesion detection rates compared with conventional imaging. 6 7 In addition, findings of hybrid positron emission tomography (PET) interphase with CT using these radiopharmaceuticals impact management decisions that lead to improved clinical outcomes of salvage therapy. 8 9

Early PCa recurrence occurs in the prostate bed, 10 which makes salvage radiotherapy to the prostate bed an effective therapy option for patients with recurrent PCa. Despite this knowledge, imaging still plays a critical role in identifying the recurrent lesion so that a higher radiation dose is delivered to the lesion. Imaging also helps identify patients with unusual sites of recurrence outside the prostate bed who may not benefit from salvage radiotherapy to the prostate bed with or without radiation to the entire pelvis. Skeletal sites of involvement are uncommon at early PCa recurrence. The bones are outside of the traditional salvage radiotherapy field, which precludes patients with skeletal metastasis from salvage radiotherapy with curative intent. Patients with skeletal metastasis are treated with systemic therapy such as androgen-deprivation therapy (ADT). The hybrid PET/CT scan provides an opportunity for molecular imaging of cancer-specific targets with PET and imaging of disease morphology with CT, both providing complementary information that improves lesion detection and characterization than either modality alone. PCa skeletal metastasis induces sclerotic bone changes, which forms the basis of the use of radionuclide bone scan for PCa imaging. PCa rarely induces osteolytic bone changes. Osteolytic bone metastasis of PCa often transitions to sclerotic lesion following successful treatment. In this report, we present a case of early PCa recurrence in the bone with an osteolytic phenotype that spontaneously transitioned to a sclerotic phenotype without treatment.

## Case Report

**Fig. 1:**
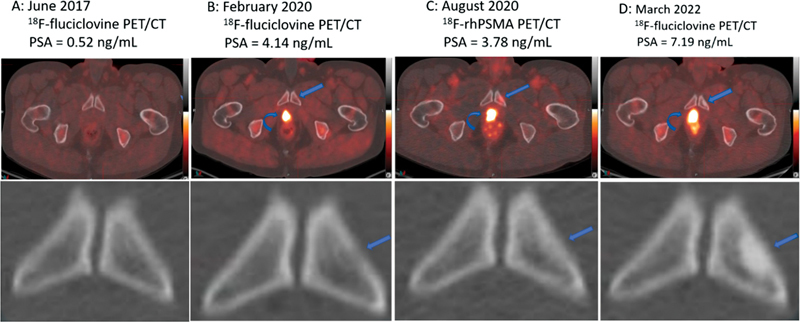
A 57-year-old man with prostate cancer recurrence had multiple time-point positron emission tomography/computed tomography (PET/CT) images showing the temporal evolution of untreated prostate cancer skeletal metastasis. ( **A** ) Fused ^18^ F-fluciclovine PET/CT ( *top plate* ) and CT image ( *bottom plate* ) were negative for the site of recurrence of prostate cancer. Specifically, no lesion is identifiable in the left pubic ramus. ( **B** ) Fused ^18^ F-fluciclovine PET/CT ( *top plate* ) shows radiotracer-avid prostate bed recurrence ( *curved arrow* ) and a subtle osteolytic lesion without radiotracer avidity in the left pubic ramus ( *straight arrow* ). The bottom image in ( **B** ) is the CT component of the PET/CT demonstrating the subtle osteolytic lesion in the inferior pubic ramus. ( **C** ) Fused ^18^ F-fluciclovine PET/CT ( *top plate* ) and CT images show the prostate bed recurrence ( *curved arrow* ) and subtle sclerosis in the left inferior pubic ramus lesion ( *straight arrows* in top and bottom images). ( **D** ) Fused ^18^ F-fluciclovine PET/CT ( *top plate* ) and CT image ( *bottom plate* ) imaging showing the prostate bed recurrence ( *curved arrow* ) and a complete osteoblastic morphology of the left inferior pubic ramus lesion without radiotracer avidity.

A 57-year-old man had a robotic radical prostatectomy in July 2014 with pathologic staging of T2cNxMx, Gleason score 4 + 3. His serum PSA level 5 months post-radical prostatectomy was 0.2 ng/mL in keeping with PSA persistence. He received 68.4 Gy to the prostate bed as adjuvant radiotherapy for PSA persistence. His nadir PSA post-radiotherapy was 0.04 ng/mL. His PSA started to rise in 2016, 0.27 ng/mL in October 2016, 0.3 ng/mL in December 2016, and 0.5 ng/mL in March 2017. In June 2017 and at a serum PSA of 0.52 ng/mL, he had an ^18^ F-fluciclovine PET/CT imaging that was negative for localization of the site of PCa recurrence ( Fig. 1A ). CT chest, abdomen, and pelvis of November 2018 and radionuclide bone scan plus CT abdomen, abdomen, and pelvis of January 2019 were also negative for the localization of the site of recurrent PCa (images not shown). In February 2020 and at a serum PSA of 4.14 ng/mL, a repeat ^18^ F-fluciclovine PET/CT was obtained, which showed an intense area radiotracer uptake in the prostate bed consistent with prostate bed recurrence of PCa (curve arrows in Fig. 1B ). In addition, there was a subtle osteolytic lesion in the left inferior pubic ramus (straight arrow in Fig. 1B ). This lesion was new and was not present on prior imaging, including the previous ^18^ F-fluciclovine PET/CT of June 2017 ( Fig. 1A ). At this time, the patient was offered systemic therapy with ADT but he declined, citing side effects concerns. However, the patient was followed up clinically.

In August 2020, the patient agreed to participate in an ongoing phase 3 clinical trial investigating the diagnostic performance of 18F Radiohybrid prostate-specific membrane antigen ( ^18^ F-rhPSMA), a novel PET radiopharmaceutical targeting PSMA, for the detection of PCa recurrence. ^18^ F-rhPSMA PET/CT done at a PSA of 3.78 ng/mL also demonstrated the radiotracer-avid prostate bed recurrence. The osteolytic lesion in the left pubic ramus shows radiotracer avidity on the fused PET/CT images (straight arrow in Fig. 1C , upper panel) and subtle sclerosis on the CT image (straight arrow in Fig. 1C , lower panel) in favor of a spontaneous transitioning of an osteolytic to osteoblastic skeletal metastasis of PCa without interval treatment. The patient continued to decline systemic therapy but was followed up regularly in the clinic. His PSA continued on an upward trajectory without treatment. In March 2022 and at a serum PSA level of 7.19 ng/mL, a repeat ^18^ F-fluciclovine PET/CT was obtained, which showed the radiotracer-avid prostate bed recurrence (curved arrow in Fig. 1D ) and dense sclerosis of the left pubic ramus lesion on CT (straight arrows in Fig. 1D ). This confirms the increased sclerosis of the left inferior pubic ramus lesion in the absence of treatment. Following this PET/CT imaging, the patient eventually agreed to commence ADT with leuprolide, which was started in March 2022.

**Fig. 2:**
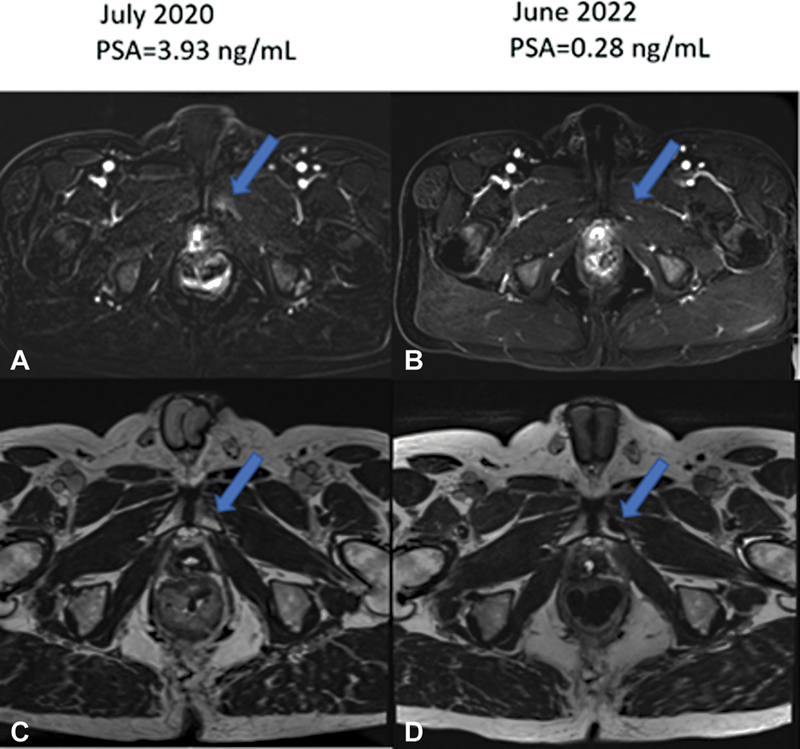
Axial slice through the pelvis before ( **A, C** ) and after ( **B, D** ) androgen deprivation therapy. In ( **A, C** ), there is restricted diffusion and enhancement ( **A** , *arrow* ) and edema on T2 ( **C** , *arrow* ) in the left inferior pubic ramus lesion. In ( **B, D** ), there is a resolution of the restricted diffusion and enhancement ( **B** , *arrow* ) with sclerosis appearing in place of edema on T2 ( **D** , *arrow* ) in the left inferior pubic ramus lesion. PSA, prostate-specific antigen.

As part of imaging for the evaluation of PCa recurrence, the patient had a pelvic MRI in July 2020 at a PSA of 3.93 ng/mL. The images show an irregular enhancing ovoid soft tissue mass within the prostate bed ( Fig. 2A ). In addition, there was an enhancing lesion in the left pubic ramus consistent with skeletal metastasis (arrows in Fig. 2A ) and congruent with findings on PET/CT imaging. In June 2022, after 3 months of ADT with leuprolide and a PSA of 0.28 ng/mL, a repeat pelvic MRI was obtained. The left pubic ramus skeletal metastasis was no longer enhancing on MRI (arrows in Fig. 2B ), indicating response to ADT and congruent with PSA decline (biochemical response to therapy). He is doing well on ADT and his most recent serum PSA level is now undetectable with a castrate level of total serum testosterone.

## Discussion

Following the excellent PCa recurrence detection rates in phase 3 trials, 11 12 radiopharmaceuticals targeting PSMA expression and amino acid trapping by PCa cells are now approved by the Federal Drug Administration for imaging of PCa recurrence. The sensitivity of these radionuclide imaging modalities for early detection of PCa recurrence has led to their application for patient imaging at very low PSA levels. Here, we present the case of a patient with biochemical recurrence of PCa following radical prostatectomy and adjuvant radiotherapy for PSA persistence. The patient had multiple PET/CT scans with radiotracers targeting amino acid trapping ( ^18^ F-fluciclovine) and PSMA expression ( ^18^ F-rhPSMA) by PCa cells. The patient had multiple PET/CT scans, the first of which was obtained at a relatively low PSA level of 0.52 ng/mL. While this first PET/CT was negative by failing to localize the site of PCa recurrence, it was useful as a normal baseline PET/CT imaging for comparison of PET/CT scans obtained subsequently. The disease evolved while the patient declined systemic treatment with ADT making it possible to track the evolution of the untreated skeletal metastasis of PCa in the patient.

The majority of PCa skeletal metastasis is osteoblastic. This is partly explained by conventional imaging (bone scan and CT) in common use that detects skeletal lesions only once cortical reaction occurs. 13 Yet, seeding of PCa cells first occurs in the bone marrow. 14 The growth of these cancer cells stimulates reactive sclerotic changes in the bone cortex. Newer sensitive molecular imaging modalities for marrow involvement such as PET have encouraged the application of imaging in earlier phases of PCa recurrence. The transformation of osteolytic or “silent on CT” to osteoblastic bone lesions in response to systemic therapy is a well-known phenomenon. This case highlights the possibility of PCa skeletal metastasis beginning as an osteolytic lesion and spontaneously transitioning to an osteoblastic lesion without treatment. Awareness of this possibility is important to avoid pitfalls in image interpretation. Dynamic crosstalk exists between cancer cells and the bone marrow microenvironment, particularly osteoblasts and osteoclasts. 15 Osteoblastic and osteolytic changes may coexist in early PCa skeletal metastasis. Osteoblastic changes occur at the edges of the tumor foci and in mature lamellar bone via appositional bone formation. 16 Osteolysis, which may alternate with bone formation, is also prominent at this stage and manifests as loss of bone trabeculae. 16 Over time, the population of osteoclasts driving osteolytic changes declines. 16 PSA has been implicated in this decline in the population of osteoclasts at the site of skeletal metastasis of PCa. 17 The decline in the population of osteoclasts relative to osteoblasts is, perhaps, responsible for the predominance of osteoblastic imaging phenotype characteristic of PCa.

Hybrid PET/CT imaging allows for the detection of skeletal metastasis in this patient, guiding the selection of the optimum therapy. About a third of all patients imaged with ^18^ F-fluciclovine PET/CT for salvage therapy planning have their treatment approached changes based on imaging findings, mostly in the form of a change from salvage radiotherapy to systemic therapy due to the detection of recurrence outside of the traditional savage radiotherapy field. 18 Management decision that is informed by ^18^ F-fluciclovine PET imaging findings is associated with improved 3-year biochemical failure-free survival even at very low serum PSA levels. 9 19 Aided by the CT-identified left pubic ramus lesion, hybrid PET/CT was helpful in selecting the optimum therapy for the treatment of recurrent PCa in this patient. ADT deprives PCa cells of the growth signal induced by androgens. Following the institution of ADT, the serum PSA declines promptly from 7.19 ng/mL in March 2023 to 0.28 ng/mL 3 months later. Correspondingly, the lesion in the left inferior pubic ramus demonstrated on an earlier pelvic MRI of July 2020 showed reduced signal intensity in congruent with biochemical response to ADT.

An interesting finding is seen in the radiotracer avidity of the left inferior pubic ramus lesion. The lesion demonstrated radiotracer avidity only on the ^18^ F-rhPSMA PET but not on any of the three ^18^ F-fluciclovine PET scans. The avidity of radiotracers is dictated by the expression of their targets in a given lesion. ^18^ F-rhPSMA targets PSMA expressed by PCa cells, while ^18^ F-fluciclovine targets amino acid transporters expressed on the PCa cell membrane. 4 5 Differential expression of PSMA and the different amino acid transporters in PCa has been recently reported. 20 PSMA expression is a marker of aggressive disease biology. 5 PCa expression of LAT2, LAT3, and ASCT2 is associated with more indolent disease (Gleason score of ≤7). 20 This differential expression in the targets for the different tracers may explain the differences in the avidity patterns seen on the PET/CT scans.

Neither osteolytic nor osteoblastic skeletal lesion is specific for PCa skeletal metastasis. In the absence of lesion biopsy for histological confirmation, a bone lesion may be due to a myriad of disease entities. In fact, fibro-osseous disease can present as a subtle osteolytic lesion with a radiolucent ground glass matrix, which may transition to an osteoblastic lesion with time. 21 The case we present here is different. In our case, we show the transition from a normal bone architecture at a PSA of 0.5 ng/mL, which transitions to a lytic lesion at a PSA of 4.14 and finally evolves into an osteoblastic lesion at a PSA of 7.19 ng/mL. This lesion is also demonstrated on pelvic MRI and its resolution is demonstrated on a follow-up MRI obtained after the institution of treatment with ADT. This pattern is most characteristic of an evolving PCa skeletal metastasis that responded to ADT. In contrast, fibro-osseous disease will most likely demonstrate an abnormality in the bone on the baseline imaging. The evolution of the morphological changes in the lesion is also highly unlikely to track consistently with disease progression (rise in serum PSA) and response to ADT (decline in serum PSA levels). This case, therefore, makes a novel contribution to knowledge by showing that PCa skeletal metastasis can start off as an osteolytic lesion and spontaneously transitions to an osteoblastic bone lesion without treatment. Previous animal work provides a scientific basis for this and the awareness of this is very helpful for physicians interpreting imaging obtained in the early phase of PCa recurrence. 16 17
